# Cell Secretion: Current Structural and Biochemical Insights

**DOI:** 10.1100/tsw.2010.193

**Published:** 2010-10-12

**Authors:** Saurabh Trikha, Elizabeth C. Lee, Aleksandar M. Jeremic

**Affiliations:** Department of Biological Sciences, The George Washington University, Washington, D.C., USA

**Keywords:** fusion pore-porosome, soluble N-ethylmaleimide-sensitive factor (NSF)-attachment protein receptors (SNAREs), calcium (Ca2+), potassium (K+), chloride (Cl-), membrane fusion, atomic force microscopy (AFM), transmission electron microscopy (TEM), electrophysiology, aquaporins, zymogen granules (ZG), synaptic vesicles (SVs), secretory vesicle swelling, cargo release, neurons, acinar cells

## Abstract

Essential physiological functions in eukaryotic cells, such as release of hormones and digestive enzymes, neurotransmission, and intercellular signaling, are all achieved by cell secretion. In regulated (calcium-dependent) secretion, membrane-bound secretory vesicles dock and transiently fuse with specialized, permanent, plasma membrane structures, called porosomes or fusion pores. Porosomes are supramolecular, cup-shaped lipoprotein structures at the cell plasma membrane that mediate and control the release of vesicle cargo to the outside of the cell. The sizes of porosomes range from 150nm in diameter in acinar cells of the exocrine pancreas to 12nm in neurons. In recent years, significant progress has been made in our understanding of the porosome and the cellular activities required for cell secretion, such as membrane fusion and swelling of secretory vesicles. The discovery of the porosome complex and the molecular mechanism of cell secretion are summarized in this article.

## INTRODUCTION

Cell secretion is a fundamental cellular event implicated in the regulation of various physiological processes, such as neurotransmission, digestion of food, hormonal control of cell and reproductive cycles, and many other life processes. Cells produce and store products, including neurotransmitters, hormones, and digestive enzymes, in secretory vesicles and, upon demand, cells release vesicle cargo through a highly regulated process of cell secretion. Regulated vesicular release involves calcium ion ($Ca^{2+}$)–triggered membrane fusion of secretory vesicles with specialized structures at the cell plasma membrane called porosomes[1] and expulsion of vesicular content into the extracellular environment[2]. Besides $Ca^{2+}$, an involvement of a specific set of cell secretory proteins, termed SNAREs (soluble N-ethylmaleimide-sensitive factor [NSF]-attachment protein receptors), is also required for the fusion of secretory vesicles with porosomes[3,4]. SNAREs and $Ca^{2+}$ have been proposed to serve as minimal membrane fusion machinery in cells[5,6]. Vesicle SNAREs (v-SNAREs) residing on vesicle membranes and target SNAREs (t-SNAREs) on the target plasma membranes (PM) interact to form a highly stable trans-SNARE complex to fuse opposing membranes and to release vesicle cargo. In many secretory cells, vesicles are recruited to the "active zones" of the target-releasing sites, but do not readily fuse in a wait for the $Ca^{2+}$ signal. While SNAREs, $Ca^{2+}$, and $Ca^{2+}$-sensing proteins (or synaptotagmins) represent a central part of the regulated vesicular release process (with the express understanding that plant cells undergo membrane fusion, but lack synaptotagmins), we also discuss here the contributions of other players to the regulatory process in cell secretion.

Over the years, the term "fusion pore" has been referred to plasma membrane dimples that originate following a secretory stimulus, or to the continuity or channel established between opposing lipid membranes during membrane fusion. The "*porosome*", however, is a permanent, supramolecular, cup-shaped lipoprotein structure at the cell plasma membrane, where secretory vesicles transiently dock and fuse to release intravesicular contents[1,8,9,10,11,12,13,14,15,16]. The SNARE-induced fusion of secretory vesicles at the porosome base involves the establishment of continuity between the opposing bilayers[9,10,14], which is conventionally referred to as "fusion pore". Hence, for clarity, the term "porosome" was assigned to this newly discovered structure at the cell plasma membrane[9,10]. In this review article, we summarize studies and discoveries that led to the elucidation of the porosome as universal cell secretory machinery and the key cellular components involved in regulated cell secretion.

## SIZE, STRUCTURE, AND COMPOSITION OF THE "POROSOME" CELL SECRETORY MACHINERY

In the mid 1990s, atomic force microscopy (AFM) performed on isolated live pancreatic acinar cells in near physiological buffer solution provided, for the first time, the structural details and topology of the cell plasma membrane at nanometer resolution and in real time. AFM revealed circular structures termed "pits" that measure, on average, 400–500 nm in diameter and contain "depressions" that measure, on average, 100–180 nm in diameter and 25–45 nm in depth[1]. When pancreatic acinar cells were stimulated to secrete, the depressions enlarged by 25–30% in diameter and relative depth, and they returned to their resting size following completion of secretion. The enlargement of porosome diameter and the increase in its relative depth after exposure to secretagogue correlated with increased cargo (amylase) secretion. Conversely, exposure of pancreatic acinar cells to cytochalasin B, a fungal toxin that inhibits actin polymerization in cells, resulted in a 15–20% decrease in porosome size and a consequent 50–60% loss in secretion[1]. These results suggested the "depressions" to be the elusive fusion pores or porosomes in acinar cells of the exocrine pancreas[1]. Subsequent studies using growth hormone–secreting cells of the pituitary gland also demonstrated the presence of porosomes and the release of growth hormone through this elegant plasma membrane structure[7]. Sequel studies on other secretory chromaffin cells, mast cells, β-cells of the endocrine pancreas, and neural cells all demonstrated the presence of permanent porosome structures at their cell plasma membranes, and depicted the difference in their size (diameter): from ~12–14 nm in neurons and astrocytes to ~150–200 nm in endocrine and exocrine cells[8,9,10,11,12,14,15]. Porosomes in secretory cells exhibit characteristic cone/cup-shaped morphology, and the presence of a central plug in neurons and astrocytes[11,12,15]. AFM analysis of the synaptic membrane preparations revealed porosomes to be cup-shaped lipoprotein structures, consisting of eight vertical units or spokes symmetrically arranged around a central channel and the central plug (Fig. 1). Similarly, eightfold rotational symmetry and the eight spokes divided into two almost identical nucleoplasmic and cytoplasmic halves that span the nuclear membrane are the important structural attributes of the nuclear pore complex (NPC)[17,18]. While both transport systems share high symmetrical organization and display several structural elements that resemble each other, such as spokes and lateral membrane rings[9,10,11,14,15,17,18], they are quite unique in their function. Whereas the nuclear pore acts as a sieve for the transport of molecules into and out of the nucleus[19], the porosome is exclusively for the regulated expulsion of intravesicular contents, resulting from the turgor pressure generated as a consequence of the rapid entry of water and ions into the secretory vesicle[20,21].

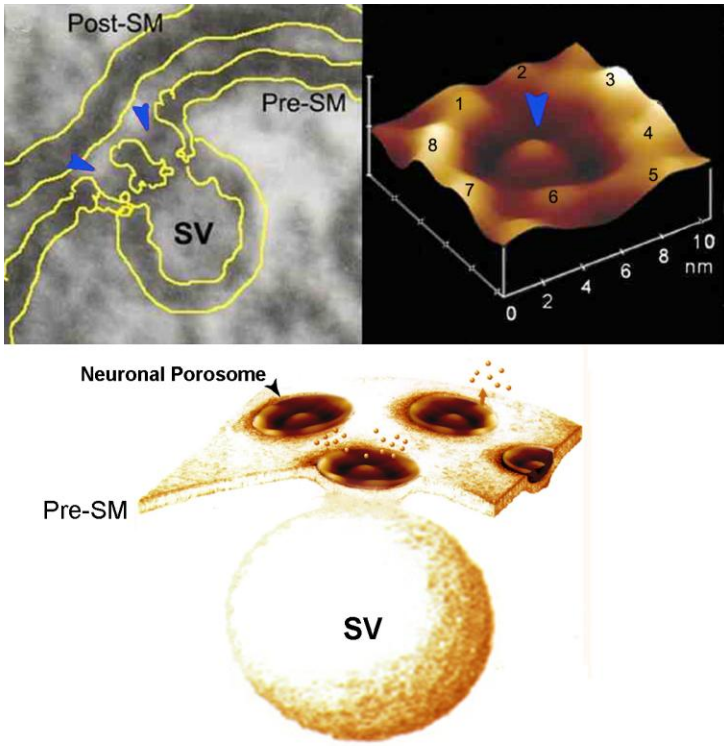

**FIGURE 1.** The neuronal porosome complex at the presynaptic membrane, where synaptic vesicles transiently dock and fuse to release neurotransmitters. The top left panel shows an electron micrograph with a 40-nm diameter synaptic vesicle fused at the base of a 12-nm cup-shaped neuronal porosome with a central plug. The top right panel depicts an atomic force micrograph of a neuronal porosome with a central plug (indicated by arrowhead). The bottom panel is a schematic illustration of native neuronal porosome complexes at the presynaptic membrane and a transiently fused synaptic vesicle at its base during neurotransmitter release. (Courtesy of Prof. Bhanu P. Jena.)

These studies collectively suggest that secretory and nuclear transport machinery in eukaryotes have evolved to transfer molecules across membranes using similar architectural, yet unique, biophysical principles, but are further tuned to accommodate the size difference among various cargo molecules. Interestingly, in nature, even simple organisms like *Toxoplasma gondii* and paramecium have developed specialized and sophisticated secretory apparatuses, such as contractile vacuoles and secretory needles, to facilitate transport of molecules across their membranes[22,23,24]. Therefore, it is not surprising that mammalian cells have developed such highly sophisticated and specialized cup-shaped supramolecular lipoprotein structures, such as the porosome complexes and the porosome-like “canaliculi system” in human platelets[25,26], for the precise and regulated docking, fusion, and release of intravesicular contents from cells. In addition to the presence of the porosome as the universal secretory machinery at the cell plasma membrane, various forms of specialized structures, such as the T-bars at the *Drosophila* synapse[27] or the “beams,” “ribs”, and “pegs” at the frog neuromuscular junction[28], have evolved, each organized to facilitate a certain specialized secretory activity in different cell types.

Further insights into the structure and function of the fusion pore complex came from reconstituted studies in which the pancreatic porosome complex was immunoisolated and then structurally and functionally reconstituted into liposomes and bilayer membranes[9,10,11]. Both AFM and transmission electron microscopy (TEM) have demonstrated that pancreatic porosomes possess a basket-like morphology, with three lateral rings and several vertically arranged ridges or spokes[9]. TEM of the pancreatic porosome complex reconstituted into liposomes revealed a 150- to 200-nm, cup-shaped, basket-like structure similar to what is observed in its native state when coisolated with zymogen granules (ZGs)[10]. To test porosome functionality, Jena's group isolated porosomes from the exocrine pancreas or whole brain, reconstituted them into the lipid membrane of the electrophysiological bilayer setup, and then exposed them to isolated ZGs or synaptic vesicles. Upon addition of secretory vesicles to one of the compartments, both the capacitance and the current of the reconstituted membrane increased[10]. This increase in the electrical activity of the bilayer was followed by the release of ZG contents (α-amylase) to the adjacent compartment. Results from these experiments confirmed that the lipid membrane–reconstituted porosomes are indeed functional[9,10,11]. Several independent studies[13,16,29,30,31,32] confirmed the presence of porosome-like structures at secretory sites and their involvement in cell secretion in various secretory cells, including pituitary cells, neurons, and retinal pigment epithelium. EM study of neurons demonstrated docking of synaptic vesicles to ~12- to 15-nm electron-dense structures of presynaptic terminals[31]. Similarly, using EM, AFM, and high-molecular-weight dyes, other laboratories have also identified porosomes in acinar cells of the exocrine pancreas[29] and on gonadotrophs of the anterior pituitary gland[30]. In agreement with earlier AFM-TEM studies of the neuronal porosome[11,32], electron tomography analysis of presynaptic sites (Fig. 2) confirmed the presence of permanent, ~15-nm, presynaptic densities to which synaptic vesicles (SVs) are attached[31]. These stable presynaptic structures could be neuronal fusion pores; however, the resolution power of electron tomography (~10 nm) impeded their full structural analysis. Nevertheless, this 3D microscopy analysis of presynaptic sites revealed the morphological constraints exerted by the presynaptic molecular scaffold: SVs are tightly interconnected in the axonal bouton and this vesicle network is preferentially connected to the presynaptic densities in the active zones (AZ).

It is well established that SNARE proteins mediate membrane fusion in cells. If porosomes are the secretory sites for secretory vesicle docking and fusion, then plasma membrane–associated t-SNARES should localize at the bottom of this structure facing cytosol. An immuno-AFM study performed on inverted, inside-out, isolated pancreatic plasma membrane preparations has indeed demonstrated the selective localization of t-SNARE pancreatic isoform SNAP-23 to the porosome base, confirming that these permanent plasma membrane structures serve as secretory sites for vesicle docking and fusion in the pancreas[9]. Other studies have further demonstrated that neuronal t-SNARE syntaxin-1 regulates the release of neurotransmitters by lining the fusion pore in neurons[33]. Taken together, these studies demonstrate porosomes to be permanent supramolecular lipoprotein structures at the cell plasma membrane, where secretory vesicles transiently dock and fuse to release vesicle cargo[34,35].

The biochemical composition of the pancreatic and neuronal porosomes, and the distribution of various porosome proteins, have been recently determined using immunoprecipitation, immuno-AFM, and two yeast-hybrid studies[9,10,11,12,36]. Porosome composition has been implied from the mechanism of action of several inhibitors of vesicular release, such as clostridial toxins (SNAREs proteases), agatoxins ($Ca^{2+}$ channel antagonists), and cytochalasin D (actin depolarization agent). For instance, pretreatment of cells with the fungal toxin cytochalasin, a known inhibitor of actin polymerization, results in the collapse of the porosome and, consequently, inhibition of cell secretion[1]. This study clearly demonstrates actin to be a major component of the pancreatic porosome complex. Similarly, it has been demonstrated that plasma membrane–associated t-SNARE syntaxins-1/2 and SNAP-23/25, which are specifically degraded by Botulinum toxins A, C1, and E[37], are integral parts of the pancreatic and neuronal porosome complexes[9,10,11]. In the pancreas, studies have shown that SNAP-23 localizes to the base (cytosolic side) of the porosome complex, where membrane-bound secretory vesicles dock and fuse with plasma membranes to release their cargo[10]. Furthermore, studies demonstrate the colocalization of voltage-dependent $Ca^{2+}$ channels (VGCCs) at release sites in a number of cells by using combined techniques of confocal imaging and electrophysiological recordings[38,39,40,41]. The association of syntaxin-1A, SNAP-25, and synaptotagmin with N-, P/Q-, and L-type VGCCs is suggested from the results of coimmunoprecipitation experiments[3,42,43,44,45,46]. *In vitro* binding studies demonstrate that presynaptic N- and P/Q-type VGCCs interact directly with two presynaptic membrane proteins, syntaxin-1 and synaptotagmin, through a specific synprint (synaptic protein interaction) site at the II-III cytosolic domain both in a $Ca^{2+}$-independent[42] and in a $Ca^{2+}$-dependent manner[45,46]. The II-III domain of the 1c L-type

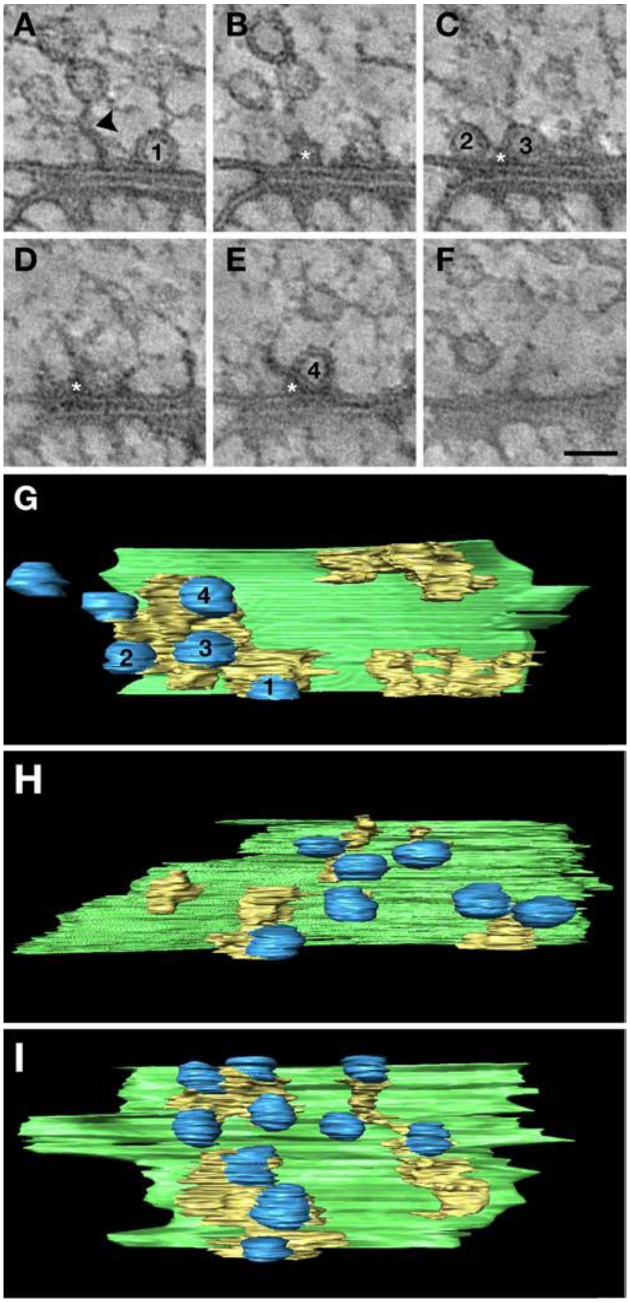

**FIGURE 2**. The three-dimensional (3D) architecture of the presynaptic cytomatrix. Association of docked SVs with presynaptic densities revealed by electron tomography. (A–F) Six virtual sections from a tomogram. The sections are separated by ~19 nm in the *z*-axis. The docked SVs (numbered 1–4) are apposed to a presynaptic electron-dense material (asterisk). A filament (arrowhead) emerging from the electron-dense material contacts an SV. (G–I) 3D reconstruction of the docked SVs (blue) and the presynaptic electron-dense material (yellow) in front of the postsynaptic density (green). G is the same synapse as in A–F. H and I are two other examples. Scale bars: A–F, 50 nm. Reprinted with permission from Siksou et al.[31]. Copyright 2007 the Society for Neuroscience.

$Ca^{2+}$-channel subunit, which is predominantly expressed in endocrine and neuroendocrine cells, also binds syntaxin-1 and SNAP-25, but with lower affinity compared to the N and P/Q channels[44]. Furthermore, studies performed in *Xenopus* oocytes demonstrate a strong functional relationship between synaptic proteins and $Ca^{2+}$ channels, since channels exhibit profoundly different kinetic properties in the presence and absence of SNAREs[44,47]. Along with functional studies, the proximity of $Ca^{2+}$ channels to the releasing sites and the interaction of $Ca^{2+}$ channels with synaptic proteins as revealed by *in vitro* binding assays further provide indirect, but strong, evidence of the physical interaction between these proteins. However, combined immunoprecipitation and two yeast-hybrid studies further revealed that the C-terminal domain of pancreatic SNAP-23 directly interacts with L-type $Ca^{2+}$ channels in pancreatic membranes[36]. Consistent with this finding, studies demonstrated that the t-SNARE neuronal isoforms, syntaxin-1 and SNAP-25, physically interact with P/Q subtypes of $Ca^{2+}$ channels in synaptic membranes[46]. These interactions are mediated by plasma membrane cholesterol[48]. Interestingly, in addition to mediating interactions of VGCCs with SNAREs[47,48,49,50,51], membrane cholesterol is also required for maintaining physical integrity and function of the porosome complex[12]. The finding that $Ca^{2+}$ channels interact with SNAREs in a cholesterol-dependent manner in many secretory cells may explain, at least in part, the corresponding inhibitory effect of cholesterol-depleting agents and clostridial toxins on regulated ($Ca^{2+}$-dependent) vesicular release[52,53,54,55,56]. These seminal findings postulate that specific protein-sterol, protein-lipid, and protein-protein interactions regulate the assembly and function of the porosome on the plasma membranes. The importance of the proximity of the $Ca^{2+}$ channel to the SNARE complex on cell plasma membranes and the role of $Ca^{2+}$ and SNARE proteins in membrane fusion are discussed further in the text.

In addition to $Ca^{2+}$ channels, SNAREs, and actin, other cytoskeletal proteins implicated in intracellular vesicle traffic have been found to associate with the porosome complex. For instance, α-fodrin, which was previously implicated in exocytosis, has been shown to interact directly with t-SNAREs[57]. Similarly, vimentin filaments interact with SNAP-23/25 to control the availability of free t-SNAREs for assembly of the t-/v-SNARE complex[58]. Collectively, these findings suggest that the cytoskeleton proteins vimentin, α-fodrin, and actin, along with SNAREs, are essential components of the porosome complex. Vesicle-associated proteins, such as v-SNAREs, synaptophysin, and myosin, may temporarily associate with the porosome when a secretory vesicle fuses with membranes. This association can occur through the globular tail domain of myosin V, which contains binding sites for VAMP and syntaxin[59,60]. Previously, the interaction of myosin V with syntaxin had been shown to require both $Ca^{2+}$ and calmodulin[60]. It was also reported that VAMP acts as a myosin V receptor on secretory vesicles and may regulate formation of the SNARE complex[57]. Furthermore, it had been shown that synaptophysin interacts with the VAMP/myosin V complex[59]. In agreement with these earlier findings, current studies demonstrate that SNAP-23/25; syntaxin-1/2; the cytoskeletal proteins actin, α-fodrin, and vimentin; and L- and N-type $Ca^{2+}$ channels, together with the SNARE regulatory protein NSF, associate with the pancreatic and/or neuronal porosome complexes[9,10,11,12]. Additionally, $Cl^-$ ion channels $ClC_2$ and $ClC_3$ have also been identified as part of both porosome complexes[10,11]. A number of other proteins and their isoforms identified as components of the porosome complex have also been reported using 2D-BAC gel electrophoresis[10]. Given the high specificity and complexity of the membrane fusion process, it is realistic to expect that the list of regulatory proteins and lipids associated with the porosome complex, including SNARE activators like synaptotagmins, Sec1, and Munc18, or inhibitors like complexins[61,62,63,64,65,66,67,68], will continue to grow.

## KINETIC, CONFORMATIONAL, AND ATOMISTIC INSIGHTS OF SNARE-MEDIATED $CA^{2+}$-DRIVEN MEMBRANE FUSION PROCESS

The first *in vitro* assay to study SNARE-mediated membrane fusion was developed by Rothman and coworkers[5]. This bulk liposome fusion assay demonstrated that SNAREs alone can fuse opposing membranes, albeit at a very slow rate ($t_{1/2}$ = 10–40 min)[5]. However, the SNARE density in the reconstituted liposomes that investigators generally used in these early bulk liposome assays is currently considered too high (10 times higher than that in native membranes) to be physiologically relevant. The first direct demonstration of a key role for $Ca^{2+}$ in SNARE-mediated membrane fusion was obtained in a study in which the authors used a similar reconstituted liposome model system, but used physiologically relevant t-/v-SNARE concentrations[6]. When neuronal t-SNARE and v-SNARE reconstituted liposomes interacted in the absence of $Ca^{2+}$, fusion between opposing t-/v-SNARE vesicles proceeded slowly, in the order of minutes ($t_{1/2}$ = ~16 min), as previously reported[5]. In the presence of $Ca^{2+}$, however, vesicles fused rapidly within seconds ($t_{1/2}$ = ~10 sec)[6]. The physiological relevance of these results can be inferred with a calculation by assuming the same rate ($k = 0.0911$ $sec^{-1}$) for fusion of liposomes in the presence of calcium[6] and for fusion of the native vesicles with the plasma membrane. With this assumption, the first 1% of vesicles from the cellular pool would fuse within 100 msec. In a neuroendocrine cell, using this same assumption, the time required for the fusion of the first vesicle with the plasma membrane, from a pool of 30,000 stored vesicles[69,70], would require less than 0.4 msec. This is consistent with the fact that, under physiological conditions, $Ca^{2+}$ entry into the synapse triggers vesicle fusion and secretion from the readily releasable pool with a delay of only 0.1–0.5 msec[71]. Hence, this simple calculation shows that $Ca^{2+}$-induced fusion of SNARE vesicles[6] is achieved in a physiologically relevant time scale. Subsequent studies confirmed the critical role of $Ca^{2+}$ and the $Ca^{2+}$-sensor protein synaptotagmin in the SNARE-mediated membrane fusion process[72,73]. Interestingly, the rate of $Ca^{2+}$-induced membrane fusion of SNARE-reconstituted vesicles obtained in this study ($k_{fus} = 0.1^{s-1}$[6]) is comparable to the rate of dissociation of SNARE-associated vesicles in the presence of NSF-ATP ($k_{diss} = 1.1^{s-1}$[74]). In contrast, fusion of SNARE-reconstituted vesicles in the absence of $Ca^{2+}$ proceeds at a much lower rate ($k_{fus} = 7 \times 10^{-4}$ $^{s-1}$[6]). Apparently, without $Ca^{2+}$, the efficient regulation of an on/off cycle of SNARE assembly and disassembly at secretory sites would not be possible. This is particularly important during the release of neurotransmitters when synaptic vesicles transiently fuse with presynaptic membranes without a loss of identity, and/or when secretory vesicles undergo multiple rounds of release and refilling cycles at AZs, a process called “kiss and run”[75,76,77]. Hence, the presence of a potent fusogen ($Ca^{2+}$) and membrane fusion inhibitor (NSF-ATP) at secretory sites provides cells with the means to synchronize SNARE-mediated membrane fusion and release of vesicular content with vesicle cycling.

Do v-SNAREs and t-SNAREs interact in the same way in solution and on membranes? Do v-SNAREs and t-SNAREs need to reside in opposing membranes in order to allow for membrane fusion? Recently, reconstituted studies have provided interesting answers to these questions[74,78,79,80,81,82]. Purified recombinant t-SNARE and v-SNARE proteins, when applied to the same lipid membranes or mica produced nonsymmetrical, globular-like cis-SNARE complexes that did not alter membrane electrical (conducting) properties[78]. In contrast, when t-SNARE vesicles were exposed to v-SNARE–reconstituted bilayers (trans-SNARE conformation), symmetrical ring-like structures were demonstrated on the membranes[74,78]. Furthermore, an electrophysiological setup revealed the fusion of t-SNARE vesicles with v-SNARE–reconstituted bilayers, which was discernible by an increase in membrane capacitance and conductivity[78]. These data conclusively demonstrated that full-length t-SNAREs and v-SNAREs need to reside and interact from opposing membranes in order to self-assemble into the physiologically relevant and fusion-competent t-/v-SNARE ring complexes. Although the crystal structure of the t-/v-SNARE complex is known[80], it should be kept in mind that atomic coordinates were determined for the postfusion cis-SNARE complex using recombinant t-SNARE and v-SNARE proteins that cannot produce functional, pore-forming complexes on membranes[74,78]. This necessitates the determination of the atomic structure of the membrane-associated trans-SNARE complex in an initial (prefusion) state. However, this remains a major challenge. Nevertheless, these structural studies provide valuable mechanistic insights into how assembly of SNARE complexes may mediate membrane fusion. Reconstituted studies further demonstrate that the disassembly of the SNARE complex, like its assembly, is a highly regulated process. The addition of the soluble NSF, an ATPase, and ATP, but not AMPPNP that is a nonhydrolyzable ATP analog, to preformed SNARE complex led to its rapid disassembly[74]. This study has shown that the t-/v-SNARE complex disassembly is an enzymatic and energy-driven process. Further analysis of NSF-ATP–induced disassembly of the SNARE complex by AFM has demonstrated NSF to function as a right-handed molecular motor[81].

The regulatory role of membranes and NSF-ATP in the assembly and disassembly of the t-/v-SNARE complex has also been recently investigated using spectroscopic approaches. Circular dichroism (CD) spectroscopy confirmed that secondary structures of membrane-associated SNAREs and SNARE complexes are different from those formed in the absence of membranes[82]. Specifically, CD spectroscopy revealed that v-SNAREs, when incorporated into liposomes, exhibit a reduced level of folding, characterized by a decrease in α-helical content. Similar to v-SNAREs, the membrane-associated t-SNAREs exhibit less helical content than in suspension. Interestingly, there is no increase in the overall level of secondary structure upon complex formation. Rather, the CD spectra of the complexes are identical to a combination of individual spectra[82]. CD data support previous AFM results, which showed that lipid is required for the proper arrangement of the SNARE proteins in membrane fusion[74,79]. In the same study, the addition of NSF to the t-/v-SNARE complex results in an increase in the unordered fraction, which authors attributed to an overall disordered secondary structure of the NSF, and not necessarily to the unfolding of the t-/v-SNARE complex. In contrast, activation of NSF by the addition of ATP almost completely abolishes all R-helical content within SNARE complexes, indicating energy-driven disassembly of SNAREs[82]. These data are consistent with reports showing spontaneous assembly (coiling or zippering) of largely structured SNARE motifs into a helical t-/v-SNARE complex during membrane fusion[83,84]. In support of CD spectroscopy and electron paramagnetic resonance (EPR) data, a recent nuclear magnetic resonance (NMR) study of full-length v-SNARE synaptobrevin in micelles revealed mostly structured conformation of its SNARE motif[85]. SNARE motifs are conserved stretches of ~70 amino acids found in all SNAREs that spontaneously intertwine (coil) to form the core complex, which is thought to provide a force for the membrane fusion. The NMR study revealed that the structure of full-length synaptobrevin in micelles is segmented and dynamic, consisting of two transient helical segments flanked by disordered regions and a third more stable helix. Transient helixes 1 and 2 comprise the SNARE motif, whereas the more stable helix 3 is the transmembrane domain[85]. Based on these varied structural and motional properties along the synaptobrevin sequence, a nucleation-propagation mechanism for trans-SNARE complex formation has been proposed: helix-1 likely forms a nucleation site, the C-terminal disordered SNARE motif may arrest SNARE folding, whereas helix-2 likely couples SNARE complex folding and fusion[85]. In concordance with this proposed mechanism of the SNARE assembly, the progressive coiling of helices from their N-terminal ends toward the C-terminal transmembrane domains has been recently demonstrated[84]. The "zippering" of SNAREs may pull the two membranes into close apposition until they fuse. However, the energy released by SNARE complexes alone may not suffice to overcome hydration, electrostatic, and other repulsion forces. A recent study demonstrates that individual, membrane-bridging SNARE complexes provide much less pulling force and energy needed for membrane fusion than previously thought[86]. Thus, the molecular machinery that drives the $Ca^{2+}$-dependent release of hormones and neurotransmitters may likely involve SNAREs, $Ca^{2+}$, and a small soluble protein, complexin, described below. Besides regulating structure and conformation of the t-/v-SNARE complexes, membranes have also been found to control complex size, which is inversely proportional to the curvature of the vesicle. Larger vesicles have smaller curvatures and, consequently, they produce larger t-/v-SNARE complexes[79]. Thus, the circular arrangement of trans-SNARE complexes assembled on membranes limits surface area during fusion of opposing membranes, resulting in a restriction of channel size[78,79].

Does the distance at which t-SNAREs and v-SNAREs from opposing membranes interact have an effect on $Ca^{2+}$-induced membrane fusion and, if yes, how? This question has been addressed by X-ray diffraction and molecular dynamics simulation studies using SNARE-reconstituted liposomes as a model system[6,87]. X-ray diffraction patterns of nonreconstituted vesicles revealed two broad peaks, the stronger one being at 3.1 Å and the weaker centered at 1.9 Å[6]. The addition of $Ca^{2+}$ and/or the incorporation of SNAREs at the vesicle membrane influenced both peaks within the 2.1- to 3.3-Å intensity range. The influence of $Ca^{2+}$ and/or SNAREs was more visible on a peak positioned at 3.1Å. The authors explained this finding in terms of an increased level of vesicle pairing and/or a decrease in the distance between apposed vesicles. Incorporation of t-SNARE and v-SNARE proteins at the vesicle membrane allowed for tight vesicle-vesicle interaction, which was demonstrated as a shift of reflection maximum from 3.1 to 2.9 Å. $Ca^{2+}$ and SNAREs worked in a concerted manner to induce a much greater increase in peak intensity in the 2.9- to 3.1-Å range, with the appearance of shoulder at 2.8 Å. Taken together, these X-ray data suggest that trans-SNARE complex formation allows opposing bilayers to come within a distance of approximately 2.8 Å[6]. Using light scattering and X-ray diffraction experiments involving SNARE-reconstituted liposomes, it has become clear that fusion proceeds on physiologically relevant time scale only when $Ca^{2+}$ ions are available between the t-SNARE– and v-SNARE–apposed bilayers[6,74]. Because $Ca^{2+}$ channels interact directly with t-SNAREs[47], and because t-SNAREs and v-SNAREs in opposing bilayers interact in a circular array to form conducting channels in the presence of $Ca^{2+}$[78,79], it would necessitate that $Ca^{2+}$ ions are present between the SNARE-apposed bilayers to allow bridging of the opposing membranes. Once $Ca^{2+}$ forms such a bridge, it can no longer hold its water shells, leading to bilayer dehydration, membrane destabilization, and fusion. In accordance with X-ray diffraction studies and the $Ca^{2+}$-bridging hypothesis, molecular dynamic simulation studies have revealed that phospholipids and $Ca^{2+}$ form DMP-$Ca^{2+}$ complexes with the consequent removal of water[87]. As a result of DMP-$Ca^{2+}$ interactions, the distance between the two DMP molecules is reduced to 2.92 Å[87], which is in agreement with the 2.8-Å SNARE-induced apposition established between opposing lipid bilayers, reported from X-ray diffraction measurements[6]. Physicochemical studies performed in the 1980s by Papahadjopoulos and others also postulated the formation of such interbilayer $Ca^{2+}$-phospholipids bridges[88,89,90].

In addition to its direct effect on phospholipid head groups, $Ca^{2+}$ may also indirectly stimulate membrane fusion via activation of $Ca^{2+}$ sensor proteins or synaptotagmins[71]. Synaptotagmins interact with SNARE complexes and phospholipids in a $Ca^{2+}$-dependent manner, which in turns triggers synaptic fusion[61,65]. Although synaptotagmin is indispensible for regulated secretion (rapid and synchronous neurotransmitter release), it is actually not required for synaptic vesicle fusion[91]. Whether or not $Ca^{2+}$ directly triggers membrane fusion, its presence is critical for regulated ($Ca^{2+}$-dependent) secretory vesicle release. It is important to note here that, apparently, $Ca^{2+}$ is not required for all membrane-fusion events in cells, such as constitutive (basal) vesicular release of hormones and neurotransmitters. Having described the catalyzing role of SNAREs and $Ca^{2+}$ in membrane fusion, one may ask the following question: Is there a physiological blocker of SNARE-mediated, $Ca^{2+}$-driven secretory vesicle fusion in cells? Recent studies demonstrate that indeed there is such a clamp or blocker termed “complexins”[64]. Complexins are 20-kDa proteins that can associate with the SNARE complex, but not with individual SNAREs. At the cell plasma membrane, complexins compete with synaptotagmins for the binding sites on assembled SNARE complexes and when $Ca^{2+}$ binds to the $Ca^{2+}$ sensor synaptotagmin, the clamp is released, thereby allowing membrane fusion to proceed[66]. It has been demonstrated that the overexpression of complexin blocks membrane fusion in cells[66]. This led to the proposal that grappling proteins, synaptotagmin and claspin, act as a toggle switch of SNARE activity, allowing for the precise timing and regulation of the secretion of hormones and neurotransmitters[92]. These studies collectively provided a molecular understanding of the $Ca^{2+}$-induced, SNARE-mediated, membrane-fusion process.

## MOLECULAR MECHANISMS OF SECRETORY VESICLE SWELLING AND VESICLE CONTENT EXPULSION

Following membrane fusion, the continuity between the secretory vesicle and the extracellular solution is established, which allows the cell to release its vesicular content. In the case of synaptic fusion, this release is immediate (<1 msec), which is needed to support the rapid process of neurotransmission[71]. The release of many hormones also takes place on a similar fast sec-time scale. The release of solutes is driven by the concentration gradient established between the lumen of the vesicle and the extracellular medium. However, the speed of neurotransmission and cell signaling obviously necessitates some form of facilitated or active transport to accelerate the release of neurotransmitters and hormones, rather than depending on an energetically favorable, but slow, passive diffusion process. Accumulating evidence suggests that vesicle swelling is the force that drives the expulsion of vesicular contents from cells[93,94,95,96,97,98,99,100,101]. In addition, it has been suggested from earlier electrophysiological measurements in mast cells[102,103,104,105] and from studies on bovine adrenal chromaffin cells[106] that secretory vesicle volume increases following stimulation of cell secretion. The dynamics of secretory vesicle swelling in live cells following a secretory stimulus was first demonstrated in acinar cells of the exocrine pancreas[107] and concomitantly in synaptic vesicles[2] using AFM. Time-lapse AFM imaging of live pancreatic acinar cells revealed the presence of docked ZGs, secretory vesicles in acinar cells, lying immediately below the surface of the apical plasma membrane[2]. Following the exposure to a secretory stimulus, the majority of ZGs within cells swelled, followed by a decrease in their size and a concomitant discharge of secretory products[2]. This study directly demonstrated two essential events: (1) intracellular swelling of secretory vesicles following stimulation of cell secretion in live cells and (2) the vesicle deflation following partial discharge of vesicular contents. Analogous to ZGs of the exocrine pancreas, the swelling of the SV was demonstrated during neurotransmitter release[2].

Hence, in light of the aforementioned, one key question remains: What is the molecular mechanism of secretory vesicle swelling? Recently, the molecular players involved in vesicle swelling in secretory cells have been identified. A direct observation of swelling of isolated ZGs in real time using AFM in combination with immunochemical analysis demonstrated the presence of chloride ($Cl^-$) and ATP-activated potassium ($K^+$) ion channels at the ZG membrane. The activities of these channels correlated with ZG swelling[108,109,110,111,112,113,114,115]. Release of ZG cargo (α-amylase) from pancreatic acinar cells required the presence of both $K^+$ and $Cl^-$ ions in the incubation medium[108,109]. The regulation of $K^+$ and $Cl^-$ ion channel activities by $G_{\alpha i3}$ protein has been demonstrated in a number of tissues[116,117,118]. In a similar manner to the regulation of $K^+$ and $Cl^-$ ion channels at the cell plasma membrane, it was demonstrated that the heterotrimeric $G_{\alpha i3}$ also regulates their channel-gating properties at the ZG membrane[119]. In this study, authors used the secretory stimulus mastoparan (Mas), an amphiphilic tetradecapeptide from wasp venom, to stimulate the GTP-ase activity of $G_i/G_o$ proteins[120,121,122]. Stimulation of G proteins is believed to occur by insertion of the peptide into the phospholipid membrane and by adopting an α-helical structure that resembles the intracellular loops of G protein-coupled receptors. An active mastoparan form,Mas-7, but not its inactive variant Mas-17, in the presence of [$^{32}$P]-labeled guanosine-5'-triphosphate (GTP) stimulated swelling and GTPase activity in ZGs, confirming the presence and activity of G proteins at the ZG membrane[119]. Similarly, Mas-7 has also been demonstrated to stimulate swelling of isolated brain synaptic vesicles[123]. Pancreatic ZGs swell rapidly in response to GTP and sodium fluoride (NaF)[119], which further postulated a G protein–mediated mechanism of secretory vesicle swelling. Following exposure of ZGs to these two G protein activators, a rapid water influx into ZGs and their concomitant swelling were observed[107,119]. As opposed to the slow diffusion by osmosis, membrane-associated water channels called aquaporins (AQP) are involved in rapid water gating in cells[124,125,126]. The presence of AQP1 at the ZG membrane[107], the presence of AQP6 and $G_{\alpha o}$ protein at the synaptic vesicle membrane[123], and their involvement in GTP-mediated vesicle water gating and swelling have been demonstrated[107,123]. The involvements of additional contributors, phospholipase $A_2$ in the swelling of ZGs and $vH^+$-ATPase in the swelling of synaptic vesicles, have been also reported[21,127]. These studies demonstrate that mechanisms of water transport across the plasma and secretory vesicle membranes are quite similar. The sequence of events leading to swelling, however, remains to be determined.

To elucidate the role of swelling in vesicle plasma membrane fusion and in the expulsion of intravesicular contents, electrophysiological ZG-reconstituted lipid bilayer fusion assays have been performed. These studies demonstrate that, following stimulation of cell secretion, ZGs within pancreatic acinar cells swell, followed by a release of intravesicular contents through porosomes[2,10]. In contrast, isolated ZGs stay swollen following GTP exposure because there is no release of the intravesicular contents[2,107,119]. Within seconds following stimulation of secretion, empty and partially empty secretory vesicles accumulate within cells[128,129]. Reconstituted studies further revealed that different ZGs within cells or in isolation undergo different degrees of swelling when exposed to a certain concentration of secretory stimulus. More importantly, these studies demonstrated for the first time that there is a direct correlation between the extent of swelling and the amount of vesicle cargo released[2]. These observations may explain the graded release of hormones and neurotransmitters from cells when exposed to varying concentrations of secretory stimuli. The generation of empty secretory vesicles could result from multiple rounds of fusions-swelling-expulsion cycles during the secretory process. Another process termed "compound" secretion[130,131,132] may also contribute to partial release of intravesicular contents and the generation of partially empty vesicles. In compound secretion, secretory vesicles fuse with each other, allowing for larger content release through the single fusion pore. Because secretory sites (porosomes) at the cell plasma membrane are limited, to overcome this limitation during massive release, certain cells may reroute secretory vesicles to compound secretion. However, even via this mechanism of compound secretion, compound vesicles that fuse transiently with the plasma membrane[29,30,75,76,77,105,133,134,135,136,137,138,139] will eventually generate empty and partially empty vesicles following cell secretion. While the prevalence and relationship between two types of vesicular release remains to be determined, it has become clear that vesicle swelling is a necessary, priming step in the SNARE-mediated release of hormones, neurotransmitters, and other secretory products.

## CONCLUSION

Studies summarized in this review highlight the importance and our current understanding of the cell secretory machinery, the porosome, how it operates at the cell plasma membranes, and the way it regulates the release of hormones and neurotransmitters from cells. Porosomes represent specialized plasma membrane structures, which are universally present in secretory cells, from exocrine and endocrine cells to neuroendocrine cells and neurons. Studies demonstrate that secretory vesicles transiently dock and fuse at the base of the porosome complex to release their cargo. Therefore, the secretory process in cells is a precisely coordinated, highly regulated, and well-orchestrated multistage event in which porosomes and secretory vesicle swelling play central roles.

## ACKNOWLEDGMENTS

This work was supported by an Institutional Research Grant (IRG-08-091-01) from the American Cancer Society to The George Washington University Cancer Institute (to AJ).
